# Transformation of a Testicular Teratoma to an Aggressive Primitive Neuroectodermal Tumor

**DOI:** 10.7759/cureus.73766

**Published:** 2024-11-15

**Authors:** Aneri Patel, Panhaneath Seng, Rashmi Verma, Jasper Zheng

**Affiliations:** 1 Medical School, University of California Davis School of Medicine, Sacramento, USA; 2 Hematology and Oncology, University of California Davis Comprehensive Cancer Center, Sacramento, USA; 3 Pathology and Laboratory Medicine, University of California Davis Health System, Sacramento, USA

**Keywords:** bone metastasis, case report, germ cell tumors, malignant transformation, nonseminomatous germ cell tumor, primitive neuroectodermal tumor, testicular teratoma

## Abstract

We present a case of a 36-year-old male found to have a nonseminomatous germ cell tumor (NSGCT) with alpha-fetoprotein levels (AFP) of 737.9 ng/mL and beta-human chorionic gonadotropin (β-HCG) of 692 IU/mL. Pathology analysis after left orchiectomy showed a mixed germ cell tumor with 20% embryonal carcinoma, 20% yolk sac tumor, and 60% teratoma. The patient's AFP levels normalized three months after surgery. Chemotherapy was proposed, but the patient declined.

Two years post orchiectomy, lytic bone lesions were discovered on a surveillance CT. A bone marrow biopsy (BMB) showed neural differentiation consistent with metastatic primitive neuroectodermal tumor (PNET) arising from the previous testicular germ cell tumor. Treatment for the PNET included vincristine, doxorubicin, cyclophosphamide, and ifosfamide/etoposide mesna. Two months later, a repeat BMB showed minimal tumor cells. The patient also underwent a tandem autologous stem cell transplant with carboplatin/etoposide conditioning and adjuvant etoposide and the subsequent PET/CT scan showed a response to the above treatment.

Clinical stage I of NSGTs can often be cured with orchiectomy; the challenge is identifying high recurrence risks. This is a unique case of NSGCT transforming to PNET, an aggressive tumor with a poor prognosis given the limited response to standard chemotherapy of platinum-based drugs. Our patient underwent chemotherapy and an autologous stem cell transplant, which proved to be effective in high-risk diseases.

There are no established guidelines for the treatment for malignant transformation of testicular teratoma into PNET. The regimen for our patient yielded promising results. Our aim is to highlight a regimen that can be utilized for this rare aggressive neoplasm.

## Introduction

Germ cell tumors (GCTs) are a group of neoplasms that arise from germ cells, typically occurring in the testes and representing one of the most common malignancies among young males aged 15 to 35, despite accounting for only 1% of all solid tumors in men [1]. These tumors comprise approximately 95% of all testicular cancers, making them a critical area of focus in the field of oncology. Nonseminomatous germ cell tumors (NSGCTs), a subtype of GCTs, are notably aggressive and account for around 5% of all testicular cancers. NSGCTs are composed of multiple histological types, with common subtypes such as embryonal carcinoma, yolk sac tumor, and teratoma, each contributing uniquely to the tumor’s behavior and treatment response [2].

In our patient’s case, the mixed germ cell tumor identified on pathology included 20% embryonal carcinoma, 20% yolk sac tumor, and 60% teratoma. Teratomas, in particular, are composed of tissue from at least two different germ layers and exhibit varying degrees of differentiation [2,3]. Unlike other GCTs, teratomas carry a unique risk of malignant transformation, evolving into highly aggressive malignancies in about 3-6% of metastatic GCT cases. This transformation frequently leads to the formation of carcinomas or sarcomas, with primitive neuroectodermal tumors (PNETs) emerging as an unusual but notable outcome [4].

Primitive neuroectodermal tumors (PNETs) are part of a group of small round blue cell tumors characterized by neural differentiation. PNETs are clinically concerning due to their poor prognosis and limited response to standard cisplatin-based chemotherapy regimens typically used for GCTs [5,6]. The transformation of a testicular teratoma into a PNET is rare and carries a high mortality rate, particularly in cases with metastatic disease.

This case is unique in that it documents the malignant transformation of a testicular teratoma into a PNET, highlighting an unusual progression and suggesting a promising treatment approach. The rarity of this transformation emphasizes the importance of identifying effective treatment regimens for similarly aggressive cases.

## Case presentation

A 36-year-old male presented to the emergency department with a six-week history of left testicular swelling, aggravated by pressure, such as when his daughter sat in his lap. Previously, the patient's physicians suspected a varicocele; however, due to the progression of swelling, he sought further evaluation. He denied any pain, fever, chills, or weight loss.

**Figure 1:**
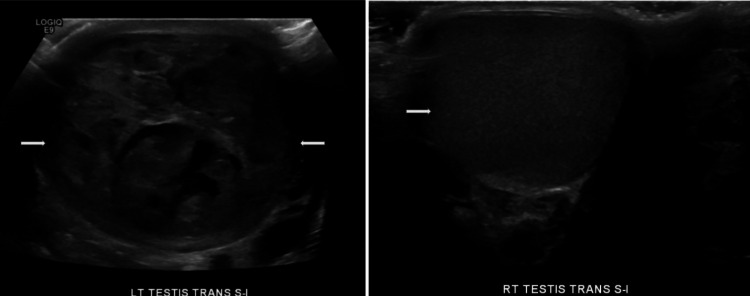
Ultrasound of the scrotum showing heterogeneous solid and cystic appearing left testicle (left) and normal right testicle (right)

Laboratory results revealed significantly elevated tumor markers, with alpha-fetoprotein (AFP) levels of 737.9 ng/mL (reference <8.8 ng/mL) and a beta-human chorionic gonadotropin (β-HCG) level of 692 IU/L (reference 0-3 IU/L). These elevated markers are critical in assessing the risk profile and aggressive nature of nonseminomatous germ cell tumors (NSGCTs). Elevated AFP and β-HCG levels are associated with a higher risk of recurrence and metastasis, influencing the initial recommendation for adjuvant chemotherapy due to the patient’s higher-risk profile. A CT scan of the abdomen and pelvis showed no evidence of metastatic disease, and an ultrasound showed a left testicular tumor with heterogeneous solid and cystic characteristics, suggestive of a large mixed germ cell tumor (Figure 1). The patient subsequently underwent a left radical orchiectomy. Pathology indicated a malignant mixed germ cell tumor, composed of 20% embryonal carcinoma, 20% yolk sac tumor, and 60% teratoma, with uninvolved margins and no involvement of the spermatic cord. The tumor measured 6 cm at its greatest dimension and showed no lymphovascular involvement.

Postoperatively, the patient's AFP levels remained elevated at 74 ng/mL, despite normalization of β-HCG and lactate dehydrogenase (LDH) levels. This delayed normalization of AFP raised concerns for residual disease, indicating a potential risk for recurrence or metastasis. This risk profile guided the recommendation for bleomycin, etoposide, and cisplatin (BEP) chemotherapy; however, the patient and his family declined, opting for close monitoring. Three months post-surgery, the AFP levels ultimately normalized, indicating no further evidence of active disease and allowing for surveillance instead of immediate chemotherapy.

**Figure 2:**
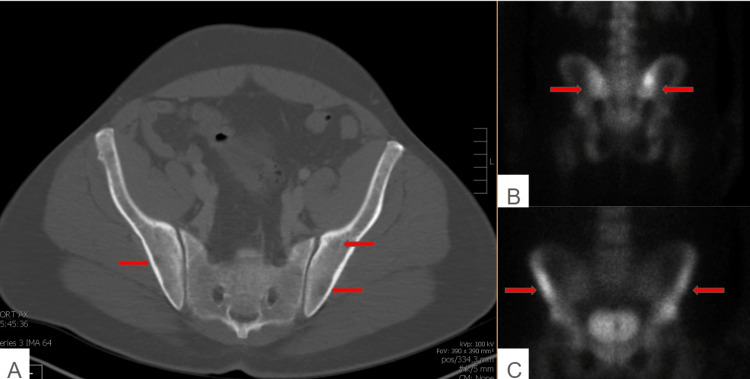
CT of the abdomen/pelvis showed a mottled appearance of the iliac crest (A). A nuclear medicine bone scan showed atypical uptake in bilateral periacetabular region (B,C) CT=Computed tomography, NM = Nuclear Medicine

**Figure 3:**
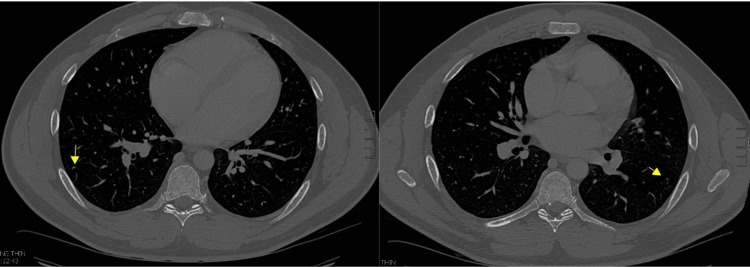
CT of the chest with contrast showed scattered tiny lung nodules CT=Computed tomography

Approximately 1.5 years post-orchiectomy, routine surveillance scans, including a CT of the abdomen and pelvis, revealed a mottled bone appearance and lytic lesions, suggestive of metastatic disease (Figure 2). A nuclear medicine bone scan demonstrated abnormal uptake in the periacetabular region and calvarium, indicating possible osseous metastasis, a finding atypical for GCTs but potentially concerning for a more aggressive, transformed malignancy. Following this scan, the patient presented with bruising and symptoms including anemia, thrombocytopenia, transaminitis, and lytic bony lesions. The patient also endorsed mild hip pain and fatigue but denied any fevers, chills, unintentional weight loss, chest pain, or shortness of breath. His vital signs were stable, and the physical examination was unremarkable. The laboratory investigation revealed an elevated LDH of 1524, while AFP and β-HCG levels remained stable. A CT scan of the head was unremarkable, and a CT of the chest showed scattered tiny lung nodules, most of which were stable (Figure 3). The ultrasound of the scrotum demonstrated a normal right testicle.

**Figure 4:**
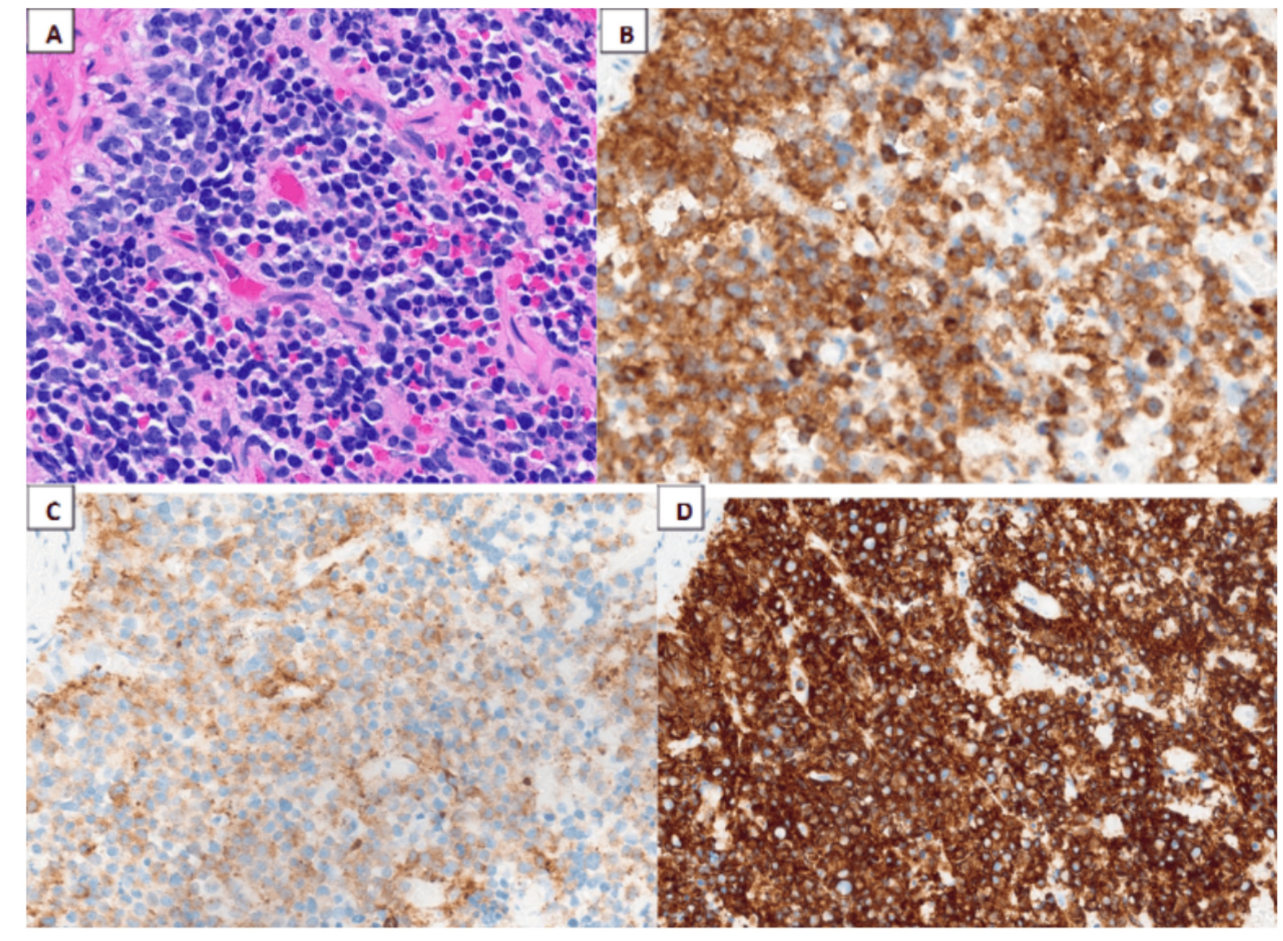
Histological examination. The histological sections showed near complete effacement of the normal hematopoietic elements of the marrow by sheets or cords of small round blue cells (A) with a high nuclear-to-cytoplasmic ratio and scant eosinophilic cytoplasm. These neoplastic cells have round nuclear contours, finely-dispersed chromatin, and one or more inconspicuous nucleoli. These small round blue cells show expression of (B) synaptophysin, (C) chromogranin, and (D) CD56.

A bone marrow biopsy (BMB) was performed to clarify the etiology of the findings, establishing a diagnosis of PNET secondary to the prior germ cell tumor. Tumor cells showed positive staining for synaptophysin, chromogranin, and CD56, while negative staining was observed for keratin, CD99, desmin, and myogenin (Figure 4). These immunohistochemical markers supported a diagnosis of small round blue cell tumor with neural differentiation, confirming PNET. 

**Figure 5:**
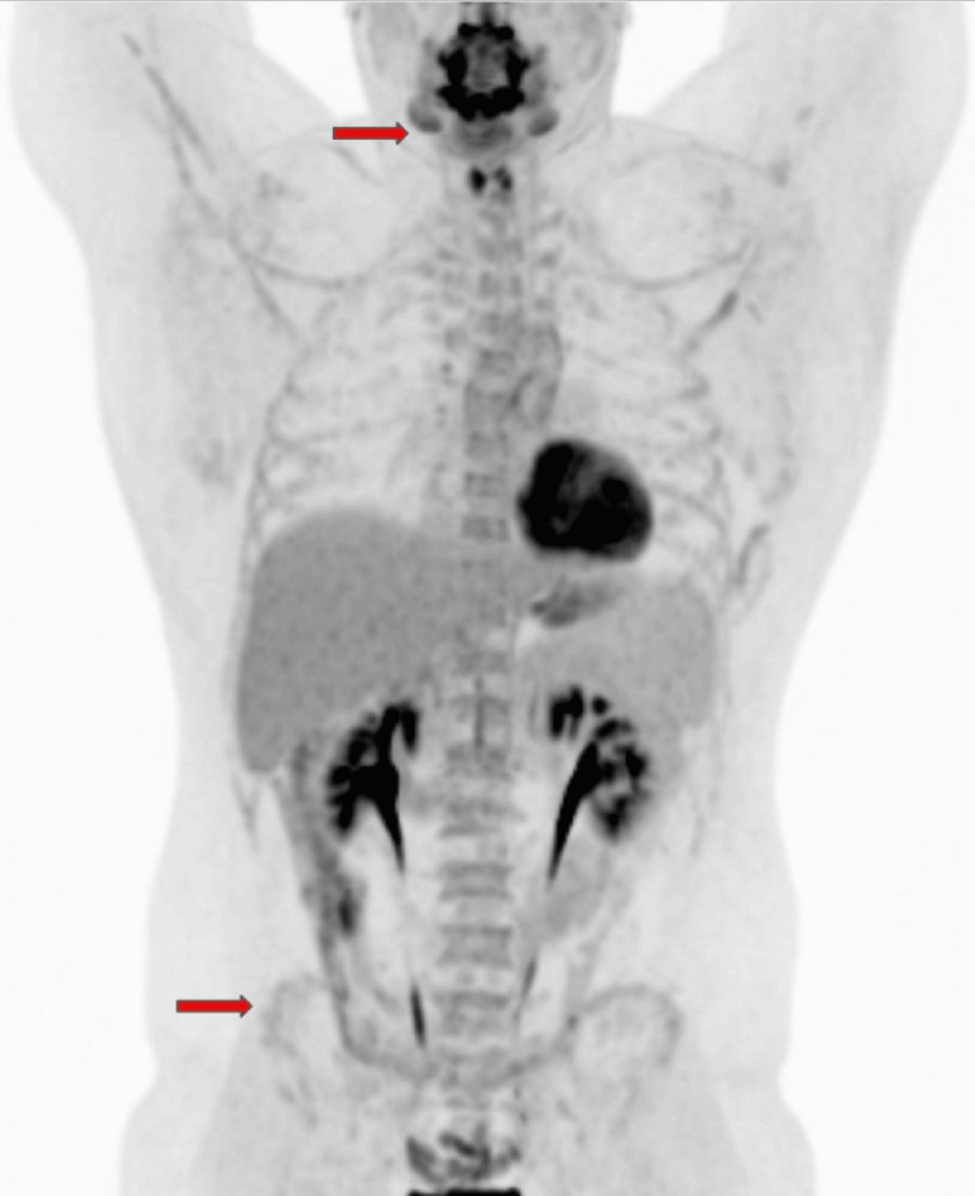
PET/CT showed a decrease in FDG avidity in the proximal appendicular skeleton. There is no FDG-avid in the cervical lymph nodes or thyroid nodule. PET/CT= Positron emission tomography/computed tomography scan, FDG=fluorodeoxyglucose

**Figure 6:**
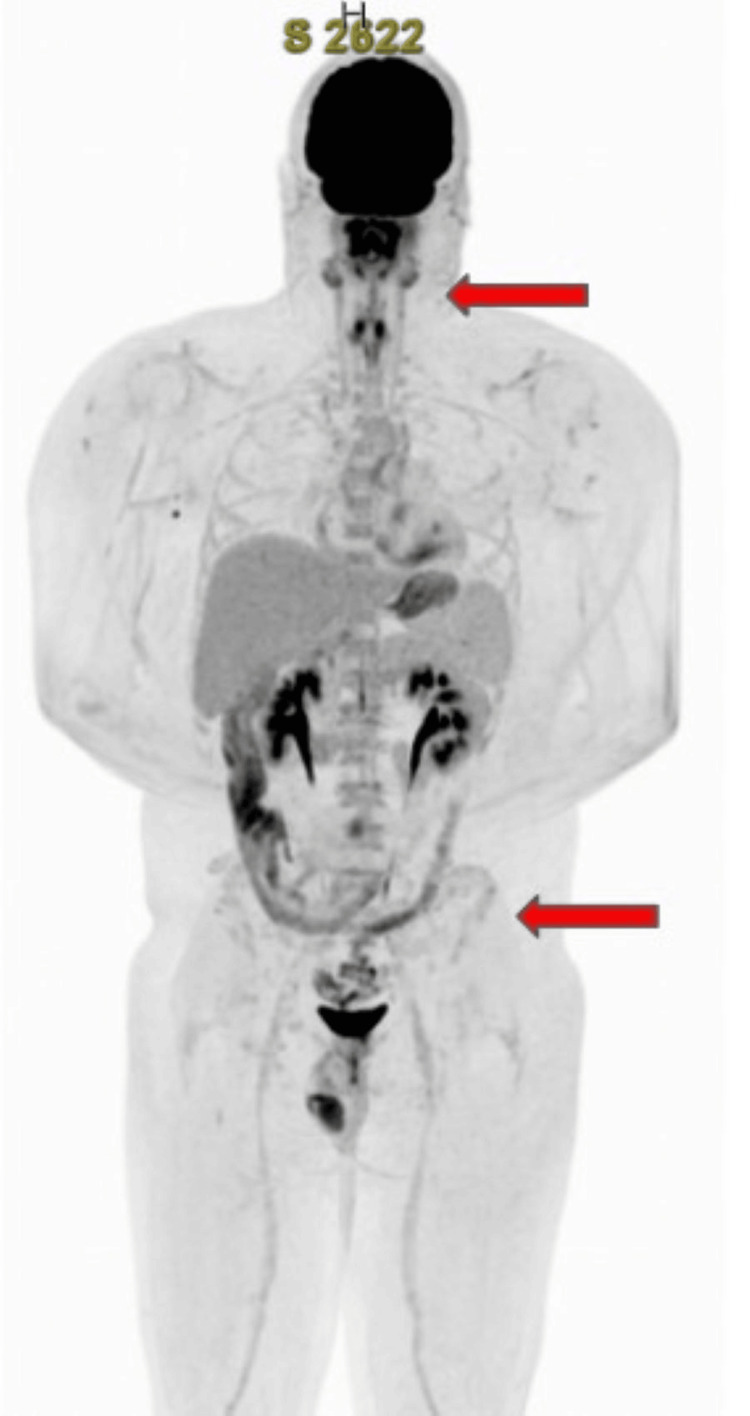
PET/CT scan after two autologous stem cell transplants showed similar lytic bone lesions without abnormal increased FDG uptake. PET/CT= Positron emission tomography/computed tomography scan, FDG=fluorodeoxyglucose

Given the rarity of this malignant transformation and the associated poor prognosis, the treatment plan involved initiating a more aggressive chemotherapy regimen with vincristine, doxorubicin, and cyclophosphamide, alternating with ifosfamide and etoposide (VDC/IE), commonly used for Ewing sarcoma and PNETs. Following this cycle, the patient demonstrated significant improvement in cell counts; however, minimal residual tumor cells remained, evidenced by the presence of rare scattered CD56, chromogranin, and synaptophysin-positive cells. A PET/CT scan indicated stable findings since the year before, with a further decrease in fluorodeoxyglucose (FDG) avidity within multiple lytic skeletal lesions, and no FDG-avid soft tissue lesions from the skull through the feet (Figure 5). An autologous transplant was considered as it could provide a better quality of life compared to 14 cycles of VDC/IE. The patient completed four cycles of chemotherapy over several months and subsequently underwent tandem autologous transplants. He has recovered well from the treatments thus far. His most recent PET/CT scan, conducted after two autologous stem cell transplants, showed a decrease in FDG avidity, indicating treatment response (Figure 6). Currently, the patient is receiving adjuvant etoposide with a treatment plan of three months (100 mg daily of etoposide for 21 days of a 28-day cycle).

## Discussion

Most testicular cancers are of the germ cell tumor (GCT) type, representing one of the most curable forms of solid neoplasms, with a survivability rate exceeding 95%. In contrast, NSGCTs account for only 5% of testicular cancers, and treatment often involves radical inguinal orchiectomy followed by chemotherapy, especially for cases with elevated serum tumor markers post-orchiectomy. The majority of patients in clinical stage I of NSGCTs are cured with orchiectomy alone; however, identifying high-risk patients who would benefit from neoadjuvant therapy remains challenging [2]. Elevated tumor markers, such as AFP and β-HCG, are associated with a higher risk of recurrence and aggressive disease behavior. In our case, the patient’s elevated AFP and β-HCG at presentation implied a high-risk profile, justifying the initial recommendation for chemotherapy. Post-orchiectomy, the delayed normalization of AFP further raised concerns for residual disease, guiding treatment considerations toward aggressive surveillance and follow-up.

A small subset of patients with teratoma undergo malignant transformation to primitive neuroectodermal tumor (PNET), a rare and highly aggressive progression of GCT, further complicating treatment. This transformation occurs in approximately 3-6% of metastatic GCT cases, predominantly leading to sarcomatous malignancies. It should be noted that while PNET has now been reclassified under “embryonal neuroectodermal tumor” per the 2016 WHO classification, the term continues to be recognized clinically [7]. In this case, a nuclear medicine bone scan and subsequent BMB were instrumental in confirming PNET. Imaging findings of abnormal uptake in the bone and lytic lesions are atypical for GCTs but raise suspicion for sarcomatous transformation. In contrast, BMB findings of neural differentiation definitively confirmed the PNET diagnosis. These diagnostic steps informed the transition to a PNET-specific treatment regimen and underscored the importance of combining imaging and histologic analysis to guide management in suspected cases of transformation.

The treatment of a PNET generally consists of surgical resection; however, cases that are not resectable are treated with chemotherapy. Unlike typical GCTs, a PNET is not responsive to standard cisplatin-based chemotherapy. A retrospective study by Al-Hader et al. found that the regimen of vincristine, doxorubicin, and cyclophosphamide, followed by ifosfamide and etoposide (CAV/IE), with a maximum of six cycles, has been effective in unresectable cases [8]. This regimen is also commonly used to treat Ewing sarcoma. Our patient, who had metastatic disease with bone marrow involvement, responded well to four cycles of this VDC/IE regimen, as evidenced by reduced disease burden on PET/CT scans. However, an unresectable PNET generally continues to bear a poor prognosis, and the limited genomic data on PNETs poses challenges for developing targeted treatments.

Emerging research suggests that targeted therapies may improve the prognosis for patients with transformed PNETs, particularly in cases with identifiable genetic mutations. For example, a recent case report describes the use of the tyrosine kinase inhibitor (TKI), Crizotinib, to target an overactive ALK pathway in a refractory PNET, with the patient showing a sustained response [4]. In our patient’s case, the chemotherapy regimen of VDC/IE combined with tandem autologous stem cell transplants demonstrated efficacy in reducing disease burden, potentially providing a viable option for cases where surgical resection is not possible. Overall, this case highlights the potential of a chemotherapy regimen combined with autologous stem cell transplant to minimize toxicity and decrease disease burden in metastatic PNET. However, further research is necessary to identify genetic variants in PNET and develop targeted treatment options for refractory and relapsed cases.

## Conclusions

Our patient, diagnosed with an NSGCT, specifically a mixed germ cell tumor with 60% teratoma characteristics, presented a rare and intriguing case. After undergoing surgery (radical orchiectomy), he experienced a transformation to a PNET 1.5 years later. Although initial tumor markers began to rise and CT scans appeared negative, bone imaging revealed atypical uptake in the bilateral periacetabular region, raising concerns for metastatic disease. The patient subsequently arrived at the emergency department with anemia, thrombocytopenia, and transaminitis, leading to the diagnosis of PNET with metastasis that was confirmed through outside pathology and bone biopsy. PNETs are known for their aggressive nature and poor prognosis, particularly due to their limited response to standard platinum-based chemotherapy. As a result, our patient began a VDC/IE chemotherapy regimen. Given the significant bone involvement, he was also evaluated for an autologous stem cell transplant to restore damaged bone marrow after high-dose chemotherapy cycles. This case exemplifies the rare transformation from teratoma to a PNET of the bone following 1.5 years of NSGCT orchiectomy. No established guidelines exist for treating malignant transformations of testicular teratomas into PNETs. However, the treatment regimen we implemented yielded promising results, as evidenced by a repeat bone marrow biopsy two months after the initial chemotherapy cycle. 

In summary, this case offers a novel insight into the management of PNETs arising from testicular teratoma, where a VDC/IE regimen combined with autologous stem cell transplantation demonstrated clinical benefit. This approach may provide an alternative strategy for similar cases, setting a precedent for future research into tailored treatment regimens that address the specific challenges posed by PNET transformation in germ cell tumors.

## References

[1] Siegel RL, Miller KD, Wagle NS, Jemal A (2023). Cancer statistics, 2023. CA Cancer J Clin.

[2] Nauman M, Leslie SW (2023). Nonseminomatous Testicular Tumors. https://www.ncbi.nlm.nih.gov/books/NBK568754/.

[3] Williamson SR, Delahunt B, Magi-Galluzzi C (2017). The World Health Organization 2016 classification of testicular germ cell tumours: a review and update from the International Society of Urological Pathology Testis Consultation Panel. Histopathology.

[4] Dunne RF, Sahasrabudhe DM, Messing EM, Jean-Gilles J, Fung C (2014). A case series of transformation of teratoma to primitive neuroectodermal tumor: evolving management of a rare malignancy. Rare Tumors.

[5] Comiter CV, Kibel AS, Richie JP, Nucci MR, Renshaw AA (1998). Prognostic features of teratomas with malignant transformation: a clinicopathological study of 21 cases. J Urol.

[6] Baldini EH, Demetri GD, Fletcher CD, Foran J, Marcus KC, Singer S (1999). Adults with Ewing's sarcoma/primitive neuroectodermal tumor: adverse effect of older age and primary extraosseous disease on outcome. Ann Surg.

[7] Moch H, Amin MB, Berney DM (2022). The 2022 World Health Organization Classification of tumours of the urinary system and male genital organs-part A: renal, penile, and testicular tumours. Eur Urol.

[8] Al-Hader AA, Jain A, Al-Nasrallah N, Einhorn LH (2015). Metastatic malignant transformation of teratoma to primitive neuroectodermal tumor (PNET): results with PNET-based chemotherapy. Am J Clin Oncol.

